# Case Report: Successful conversion and salvage resection of huge hepatocellular carcinoma with portal vein tumor thrombosis and intrahepatic metastasis via sequential hepatic arterial infusion chemotherapy, lenvatinib plus PD-1 antibody followed by simultaneous transcatheter arterial chemoembolization, and portal vein embolization

**DOI:** 10.3389/fimmu.2023.1285296

**Published:** 2023-10-18

**Authors:** Xin Luo, Rui-zhi Chang, Dong Kuang, Mingming Yuan, Gan-xun Li, Bixiang Zhang, Yan-jun Wang, Wan-guang Zhang, Ze-yang Ding

**Affiliations:** ^1^ Hepatic Surgery Center, Clinical Medicine Research Center for Hepatic Surgery of Hubei Province, and Hubei Key Laboratory of Hepato-Pancreatic-Biliary Diseases, National Medical Center for Major Public Health Events, Tongji Hospital, Tongji Medical College, Huazhong University of Science and Technology, Wuhan, Hubei, China; ^2^ Department of Pathology, National Medical Center for Major Public Health Events, Tongji Hospital, Tongji Medical College, Huazhong University of Science and Technology, Wuhan, Hubei, China; ^3^ Geneplus-Beijing, Beijing, China; ^4^ Department of Hepatobiliary and Pancreatic Surgery, The Second Affiliated Hospital, Fujian Medical University, Quanzhou, Fujian, China

**Keywords:** hepatocellular carcinoma, conversion therapy, hepatic arterial infusion chemotherapy, portal vein embolization, immune therapy

## Abstract

**Background:**

Advanced hepatocellular carcinoma (HCC) shows poor prognosis. Combined hepatic artery infusion chemotherapy (HAIC) and lenvatinib and PD-1 antibody therapy show promising effects in treating advanced HCC, and salvage hepatectomy further promotes the overall survival in patients who were successfully converted after combined therapy. However, salvage major hepatectomy is not always amenable due to insufficient future liver remnant volume (FLV).

**Case presentation:**

We report the case of a 59-year-old man with a huge HCC as well as multiple intrahepatic foci and portal vein tumor thrombosis at his right hemi-liver. Genomic and pathologic analyses of HCC tissue revealed a TMB-high, TPS, and CPS-high cancer, with mutated DNA damage repair gene FANCC. These results suggested that this patient may benefit from chemotherapy and immunotherapy. Thus, he received combined HAIC, lenvatinib, and PD-1 antibody treatment and showed a quick and durable response. After successful downstaging, this patient was evaluated as not suitable for salvage hepatectomy due to the low FLV. He then received simultaneous transcatheter arterial chemoembolization (TACE) and portal vein embolization (PVE). The FLV increased to meet the criteria of salvage hepatectomy. Finally, this patient underwent right hemi-hepatectomy without any severe perioperative complications. In addition, no tumor recurrence occurred during the 9-month follow-up period after surgery.

**Conclusion:**

Combined HAIC, lenvatinib, and PD-1 antibody therapy, followed by simultaneous TACE and PVE, is a safe and effective conversion therapy that promotes tumor necrosis and increase FLV in patients with advanced HCC.

## Introduction

Hepatocellular carcinoma (HCC) is a global health problem with increasing incidence and mortality, and patients with advanced HCC have poor prognosis (1). Combined intra-arterial and systemic therapies have shown efficacy in advanced HCC (2, 3). Recent studies have found that salvage hepatectomy in HCC patients who were successfully downstaged and converted by combined intra-arterial and systemic therapies could improve long-term survival (4, 5). However, in patients with huge or multiple HCC after successful conversion, sequential major hepatectomy is usually not implemented due to insufficient future liver remnant volume (FLV) and the increased risk of postoperative hepatic failure (PHLF). Portal vein embolization (PVE) is an effective bridge therapy to increase FLV before major hepatectomy. However, in HCC patients receiving combined intra-arterial and systemic therapies, the safety and efficiency of simultaneous transcatheter arterial chemoembolization (TACE) and PVE in inducing compensatory hyperplasia of the non-embolized future liver remnant has not been previously reported and still needs investigation.

Herein, we report a case of a huge HCC with portal vein tumor thrombosis (PVTT) and intrahepatic metastasis that received combined hepatic artery infusion chemotherapy (HAIC) and systemic therapies including lenvatinib and PD-1 antibody, followed by simultaneous TACE and PVE. Finally, the patient was successfully converted to radical resection, showing no evidence of recurrence during follow-up after surgery.

## Case report

**Figure 1:**
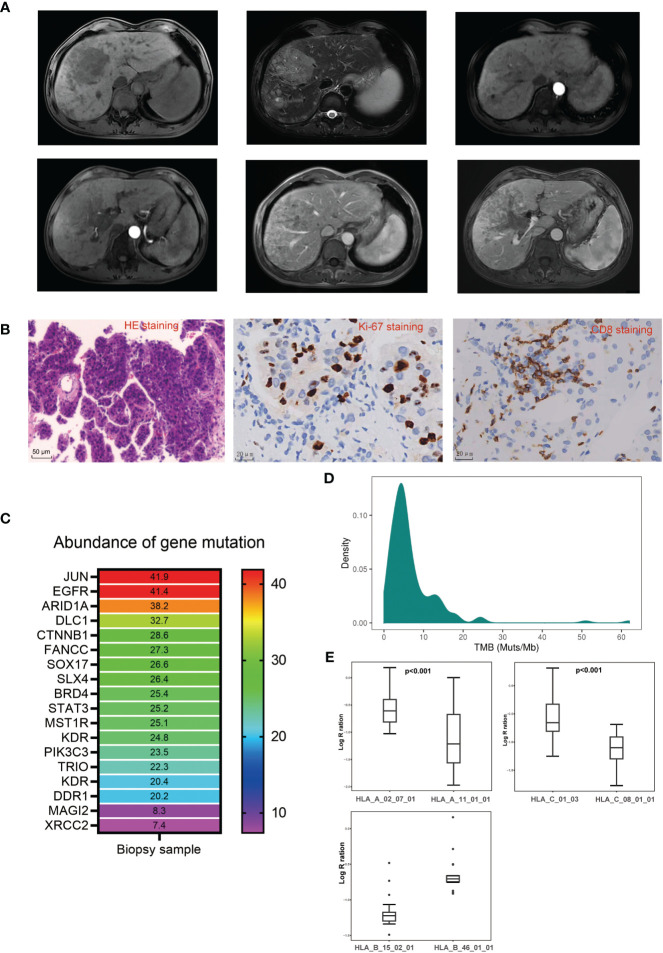
Clinical, pathological, and genetical characteristics of the patient before treatment. **(A)** Representative magnetic resonance imaging of the lesions before treatment. **(B)** Representative pathological images of the biopsy tumor tissue before treatment. **(C)** Abundance of gene mutation of the biopsy tumor tissue before treatment. **(D)** Tumor mutation burden distribution in liver cancer patients sequenced in Geneplus. **(E)** Coverage comparison of HLA-A supertype, HLA-B supertype, and HLA-C supertype.

A 59-year-old man, who was a hepatitis B virus (HBV) carrier, was diagnosed with HCC and admitted to our hospital in June 2022. He reported abdominal distension. The physical examination was normal. No family or genetic history was found. He had not received any therapy prior to admission. Magnetic resonance images revealed a huge tumor (10*7 cm) in the right lobe of the liver with multiple intrahepatic metastases, and with tumor thrombosis growing into the right portal vein (**Figure 1A**). Biochemical examinations revealed alpha-fetoprotein (AFP): >60,500 ng/mL, PIVKA-II: 18725.87 mAu/mL, and liver blood tests showed normal liver function with Child–Pugh score of 5 (grade A). With the consent of the patient, a biopsy of tumor tissue was performed. Pathologic analysis of tumor tissue confirmed the diagnosis of HCC, as indicated by Glypican3(+), CK19(−), and CK7(−), and showed poor differentiation (Edmondson grade III), and proliferative phenotype (Ki-67 Li: 50%–60%) (**Figure 1B**). A 1,021-gene panel (**Supplementary Table 1**) next-generation sequencing (GenePlus OncoD, Geneplus, Beijing) was used to analyze the gene feature of tumor tissue. The results showed that 20 genes mutated or had copy number variation in tumor tissues (**Figure 1C**, **Supplementary Tables 2, 3**). Among these mutated genes, there were some clinically significant pathogenic gene variations, such as CTNNB1 and ARID1A mutation, and MYC amplification. FANCC, a DNA damage repair (DDR)-associated gene, was also found to be mutated. No events were observed in STK11, KEAP1, MDM2/4, DNMT3A, JAK1, JAK2, JAK3, CCND1/FGF3/4/19, or PTEN, which are associated with acquired resistance or hyper-progression (HPD) to immune checkpoint inhibitors (ICIs). Tumor mutational burden (TMB) was calculated as 19.2 Muts/Mb and was classified as high TMB (**Figure 1D**). We then estimated the loss of heterozygosity (LOH) of human leukocyte antigen (HLA) class I at the somatic level and found that this patient harbored subclonal LOH of HLA-A and HLA-C supertype while maintaining HLA-B supertype heterozygosity (**Figure 1E**). Moreover, CD8 staining showed scattered infiltration of CD8+ T cells in the HCC tissue (**Figure 1B**), and PD-L1 staining revealed a tumor proportion score (TPS) of 5% and a combined proportion score (CPS) of 8.

**Figure 2:**
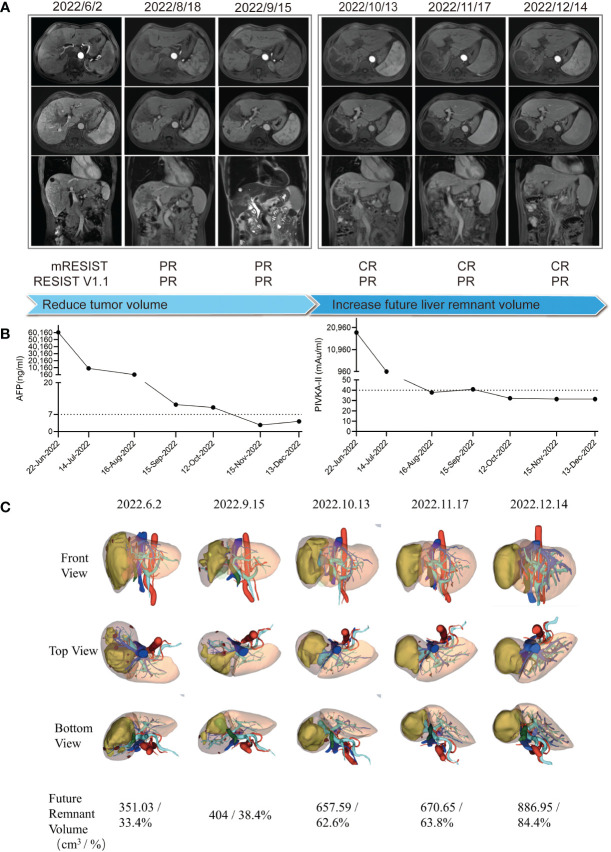
Clinical course and radiologic images throughout the conversion therapy. **(A)** Clinical course and magnetic resonance imaging changes of the lesion throughout the conversion therapy. **(B)** Tumor biomarker AFP and PIVKA-II changes throughout the conversion therapy. **(C)** 3D reconstruction model of the liver and future liver remnant volume changes throughout the conversion therapy.

After the multidisciplinary team (MDT) discussion (**Supplementary Table 5**), this patient was confirmed as both technically and oncologically unresectable HCC due to its low FLV (as shown in **Figure 2C**, the initial FLV was 351.03 cm^3^ and the standard liver volume was estimated as 1,049.86 cm^3^. The FLV estimated standard liver volume ratio (FLV/ESLV) was 33.4%), as well as its staging of BCLC stage C, CNLC stage IIIB and PVTT classified Vp3, or Cheng’s type II. However, pathological and genetic analyses indicated its potential sensitivity to ICIs, and the patient was in a generally good condition with an ECOG score of 0, and his HCC lesions located in hemi-liver without cirrhosis; thus, systemic therapy combined with local therapy was recommended. In detail, the patient received HAIC with modified FOLFOX regimen (day 1: oxaliplatin 85 mg/m^2^, leucovorin 400 mg/m^2^, and 5-fluorouracil 400 mg/m^2^ via intra-arterial infusion; days 2–3: 5-fluorouracil 2,400 mg/m^2^ via continuous intra-arterial infusion, repeated every 3 weeks) (6), lenvatinib (8 mg once daily), and anti-PD-1 monoclonal antibody (sintilimab, 200 mg intravenously every 3 weeks). In addition, he received tenofovir disoproxil fumarate 300 mg orally once a day to inhibit the replication of HBV. At the first follow-up in July 2022, blood tests revealed significant decrease in serum levels of AFP (9164 ng/mL), PIVKA-II (967.41 mAu/mL), and HBV-DNA < 1×10^2^ IU/mL (**Figure 2B**). The ultrasound showed that the diameter of the main tumor is 9.6*4.9 cm. In Aug 2022, before the third cycle of combined treatment, radiologic measurements of lesions revealed a partial response (PR) according to the modified Response Evaluation Criteria in Solid Tumors (mRECIST) criteria (**Figure 2A**). The time to response is around 6 weeks. In September 2022, the patient had undergone three cycles of combined therapy, and radiologic measurements of lesions revealed PR (mRECIST criteria), with the tumor and PVTT shrinking significantly (**Figure 2A**). Consistently, serum biomarkers of HCC including AFP (11 ng/mL) and PIVKA-II (40.96 mAu/mL) decreased significantly. In addition, serum HBV-DNA remained at low levels and liver function remained at Child–Pugh grade A (**Figure 2B**). Considering that all HCC lesions of this patient located in hemi-liver and shrunk after combined therapy, we evaluated the resectability by measuring FLV and indocyanine green retention test after 15 min (ICG-R15). Disappointingly, although ICG-R15 (5.2%) met the safety criteria for hepatectomy, the FLV was insufficient even after tumor shrinkage (as shown in **Figure 2C**, the FLV was 404 cm^3^ and the standard liver volume was estimated as 1,049.86 cm^3^. FLV/ESLV was 38.4%).

To increase the FLV of patient before the salvage hepatectomy, we implemented drug-eluting beads TACE (DEB-TACE, 40 mg pirarubicin loaded) and PVE after discussion in the MDT meeting. Lenvatinib, sintilimab, and tenofovir disoproxil fumarate were given as before. The regeneration curve (**Supplementary Figure 1**) after simultaneous TACE and PVE in this patient showed a non-linear pattern characterized by three phases: (i) initial rapid growth within 4 weeks post simultaneous TACE and PVE, (ii) a plateau, 5–9 weeks after simultaneous TACE and PVE, (iii) steady regrowth thereafter (7). Simultaneous TACE and PVE induced absolute FLV increment by 62.7% or FLV/ESLV increment by 24.2% in the initial 4 weeks of rapid growth phase. The rate of hypertrophy is 9.05 cm^3^ per day, with a 2.23% increment in FLV per day or an 0.86% increment in FLV/ESLV per day. In the steady regrowth phase, it induced absolute FLV increment by 32.2%, or FLV/ESLV increment by 20.6% within 4 weeks. The rate of hypertrophy is 7.72 cm^3^ per day, with a 1.15% increment in FLV per day or a 0.74% increment in FLV/ESLV per day (**Supplementary Table 4**). At the follow-up of 9 weeks (November 2022) after combined DEB-TACE and PVE, the tumor continued to shrink and the future liver remnant volume increased to 670.65 cm^3^, which occupied 63.8% of the standard liver volume, with the atrophy of the right hemi-liver (**Figures 2B, C**). In addition, serum levels of AFP (2.69 ng/mL) and PIVKA-II (31.5 mAu/mL) reduced to the normal range. Liver function tests showed Child–Pugh A grade and normal ICG-R15.

**Figure 3:**
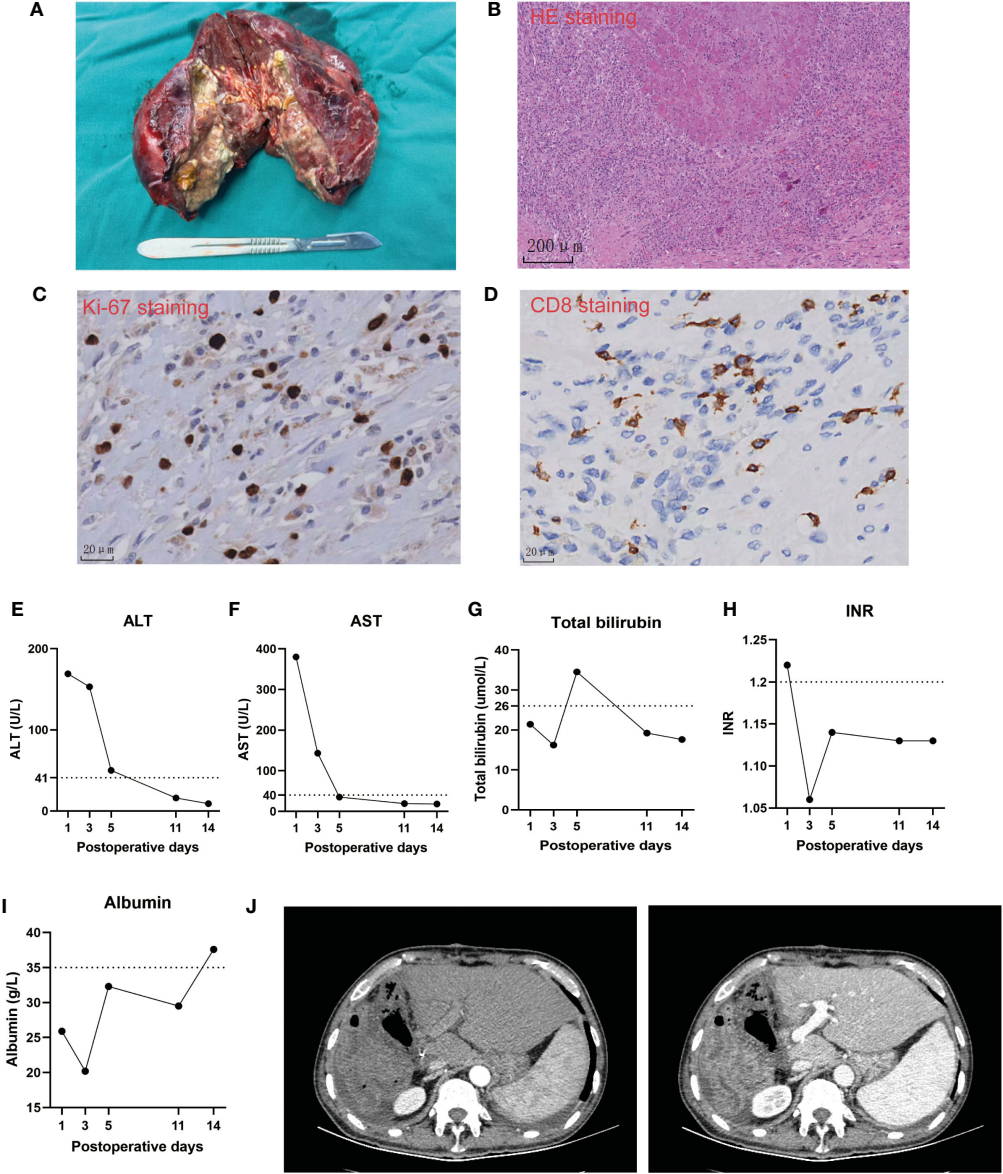
Intraoperative and postoperative clinical course of the case. **(A)** The image of the resected specimen. **(B–D)** Representative pathological images of the resected tumor. (**E–I).** Postoperative changes of biochemical tests of liver function. **(J)** Representative computed tomography images of the liver remnant after the operation.

We considered that this patient met the criteria of salvage hepatectomy according to the Chinese expert consensus on conversion therapy for HCC (8). He underwent right hemi-hepatectomy after discontinuation of lenvatinib for 3 weeks in December 2022 (**Figure 3A**). The operation time is 6 h with intermittent Pringle maneuver of 2 cycles. Intraoperative blood loss was 500 mL, and he received intraoperative transfusion of 400 mL red blood cell and 300 mL plasma. The pathological examination showed necrosis of a massive HCC (major pathological response, MPR) (9) with no tumor cells observed on the resection margins (**Figure 3B**). The immunohistochemical analysis of resected tissue showed CD8 (scattered lymphocyte+) and Ki-67 (Li: 10%) (**Figures 3C, D**). The patient only had postoperative ascites (Clavien–Dindo classification grade II) and recovered after diuretic treatment. He achieved a textbook outcome after hepatectomy and was discharged 14 days after surgery (**Figures 3E–J**).

**Figure 4:**
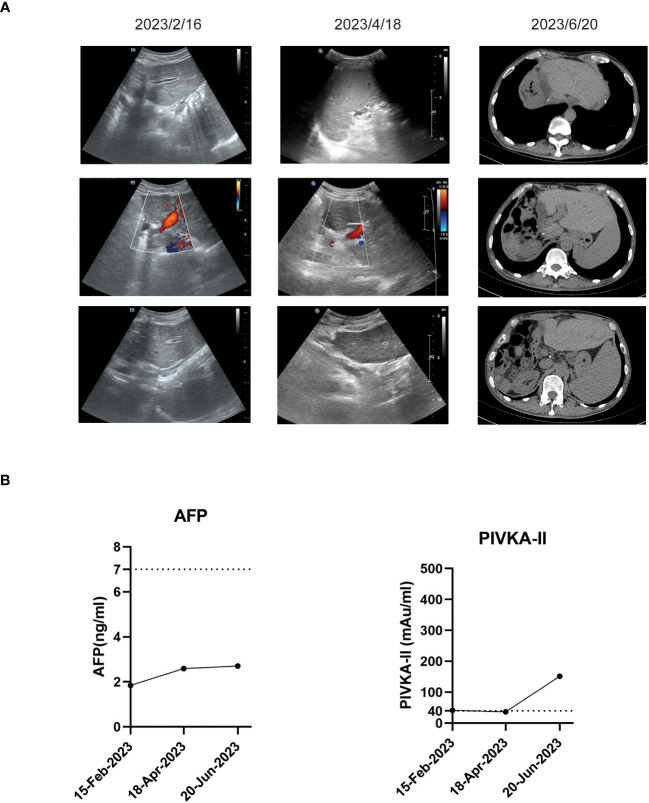
Imaging examinations and tumor biomarker changes during the follow-up. **(A)** Representative ultrasound images or computed tomography images during the follow-up. **(B)** Tumor biomarker AFP and PIVKA-II changes during the follow-up.

In the patient’s postoperative maintenance therapy, he was given lenvatinib 8 mg orally once a day, sintilimab 200 mg intravenously once every 3 weeks, and tenofovir disoproxil fumarate 300 mg orally once a day. There was no clinical evidence of recurrence at 9 months after the resection, and the AFP levels remained within the normal limit (**Figure 4**). He was still in the follow-up after surgery.

## Discussion

Combined locoregional and systemic therapy showed a promising effect in treating advanced HCC, whereas the priority population that was sensitive to the combined therapy is still elusive. In this study, we reported an HCC patient with main tumor diameter of 10 cm, multiple intrahepatic foci, and portal vein tumor thrombosis that was sensitive to the combined HAIC and systemic therapy including lenvatinib and PD-1 antibody. Genomic and pathologic analyses of HCC revealed high TMB, high CPS, and TPS in PD-L1 staining, which suggested this patient may be sensitive to the PD-1 antibody-based therapy. In addition, mutational analysis showed that tumor cells had a FANCC mutation, which was reported as sensitive markers to chemotherapy targeting DNA replication, and ICIs. Interestingly, CTNNB1 mutation, which was associated with resistance to immunotherapy in HCC (10), was also found in the HCC tissue. Considering that the high incidence (35%) of CTNNB1 mutation in HCC and co-alterations of genes were commonly found in HCC with CTNNB1 mutation; our results suggested that a subpopulation of patients with CTNNB1 mutation might be sensitive to combined immunotherapy, especially in those with high TMB, high TPS, and CPS, and co-alteration with chemotherapy- or immunotherapy-sensitive gene alterations.

PVE has been used to increase FLV before major hepatectomy, with its success rate of 60%–80%, and postoperative complication rates of 10%–20% (11). The hyperplasia of the remaining liver parenchyma after PVE takes a relatively long time (usually 4–6 weeks). In addition, more than 20% of patients lose the chance of surgery due to tumor progression or insufficient remaining liver hyperplasia (12). For these patients, the current treatment strategies include combination of TACE to control tumor progression and further promote future liver remnant hyperplasia. Previous studies reported that compared with PVE alone, TACE combined with PVE can achieve a significant increase of FLV, and a significant higher rate of complete tumor necrosis associated with longer recurrence-free survival (13), and simultaneous TACE and PVE were reported as a safe and better approach compared with sequential TACE and PVE in increasing the FLV (14). However, all these studies were performed in patients without systemic therapy, and there was no study reporting the efficacy of simultaneous TACE and PVE in increasing the FLV of HCC patients undergoing locoregional or systemic anticancer therapy. In this patient, we reported, for the first time, that simultaneous TACE and PVE effectively increased the FLV in a patient undergoing combined therapy of HAIC, lenvatinib, and PD-1 antibody, and we found that HAIC plus systemic therapy did not have a negative impact on the hyperplasia of the future liver remnant when compared with the previously reported patients who did not receive any anticancer therapy before PVE (15). Altogether, these results showed evidence that simultaneous TACE and PVE following combined HAIC, lenvatinib, and PD-1 antibody therapy is safe and efficient in increasing FLV.

## Conclusion

Combined therapy of HAIC, lenvatinib, and PD-1 antibody showed quick and durable efficacy in an advanced HCC patient with high TMB, high CPS, and TPS in PD-L1 staining. In addition, simultaneous TACE and PVE is an effective strategy to increase FLV in HCC patients who were successfully downstaged after combined locoregional and systemic anticancer therapy.

## Data availability statement

The original contributions presented in the study are included in the article/**Supplementary Material**. Further inquiries can be directed to the corresponding authors.

## Ethics statement

The studies involving humans were approved by the Ethics Committee of Tongji Medical College. The studies were conducted in accordance with the local legislation and institutional requirements. The participants provided their written informed consent to participate in this study. Written informed consent was obtained from the individual(s) for the publication of any potentially identifiable images or data included in this article. Written informed consent was obtained from the participant/patient(s) for the publication of this case report.

## Author contributions

XL: Formal Analysis, Writing – original draft, Writing – review & editing. R-ZC: Resources, Writing – review & editing. DK: Resources, Writing – review & editing. MY: Data curation, Writing – review & editing. G-XL: Writing – review & editing. BZ: Writing – review & editing. Y-JW: Conceptualization, Supervision, Writing – review & editing. W-GZ: Conceptualization, Supervision, Writing – review & editing. Z-YD: Conceptualization, Supervision, Writing – review & editing.

## Supplementary material

Supplementary Figure 1 Volume changes in future liver remnant after simultaneous transcatheter arterial chemoembolization and portal vein embolization.
