# Supplementary figures and images for: Case Report: Successful conversion and salvage resection of huge hepatocellular carcinoma with portal vein tumor thrombosis and intrahepatic metastasis via sequential hepatic arterial infusion chemotherapy, lenvatinib plus PD-1 antibody followed by simultaneous transcatheter arterial chemoembolization, and portal vein embolization
Source: Front Immunol. 2023 Oct 18;14:1285296. doi: 10.3389/fimmu.2023.1285296 (PMC10622745; doi:10.3389/fimmu.2023.1285296)

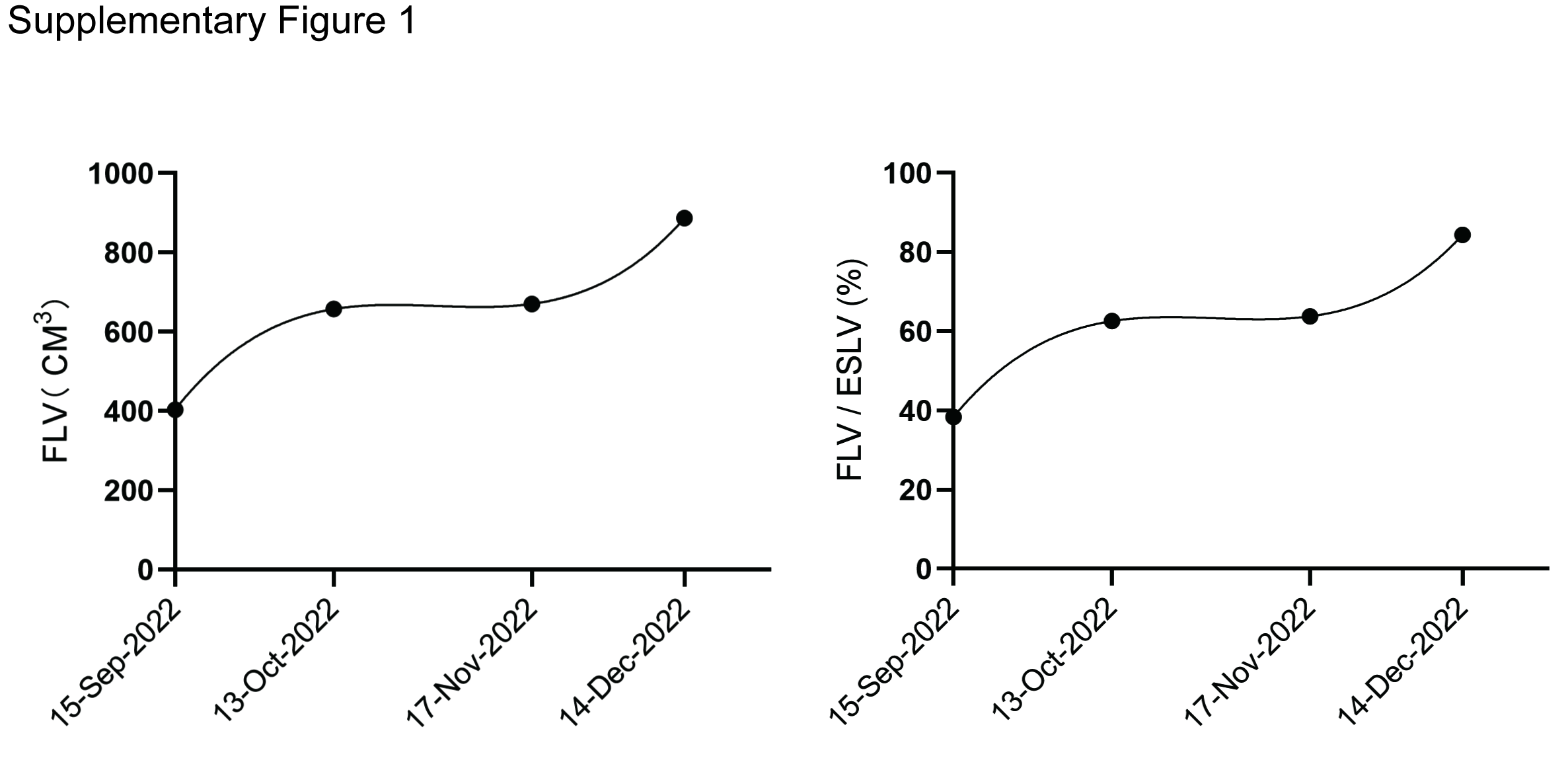

Supplement: Supplementary Figure 1 — Volume changes in future liver remnant after simultaneous transcatheter arterial chemoembolization and portal vein embolization. [file Image_1.tif]
