# Supplementary material for: Case Report: Successful conversion and salvage resection of huge hepatocellular carcinoma with portal vein tumor thrombosis and intrahepatic metastasis via sequential hepatic arterial infusion chemotherapy, lenvatinib plus PD-1 antibody followed by simultaneous transcatheter arterial chemoembolization, and portal vein embolization
Source: Front Immunol. 2023 Oct 18;14:1285296. doi: 10.3389/fimmu.2023.1285296 (PMC10622745; doi:10.3389/fimmu.2023.1285296)
Supplement: Supplementary file 2 [file Table_1.docx]

Supplementary Table 1. 1021-gene panel list

| gene | gene | gene | gene | gene | gene | gene | gene | gene | gene |
| --- | --- | --- | --- | --- | --- | --- | --- | --- | --- |
| ABCA13 | ABCB1 | ABCC1 | ABCC2 | ABCC11 | ABCG2 | ABL1 | ABL2 | ACACA | ACIN1 |
| ACTB | ACTG1 | ACTG2 | ACVR1B | ACVR2A | ACVRL1 | ADAM29 | ADAMTS5 | ADCY1 | AFF2 |
| AFF3 | AHNAK | AKAP9 | AKT1 | AKT2 | AKT3 | ALB | ALK | ALOX12B | AMOT |
| ANGPT1  ARAP3 | ANK3  ARFGEF1 | ANKRD11  ARFGEF2 | ANKRD30A  ARFRP1 | ANKRD30B  ARHGAP26 | APC  ARHGAP29 | APEX1  ARHGAP35 | APOBEC3B  ARID1A | AR  ARID1B | ARAF  ARID2 |
| ARID4B | ARID5B | ARNT | ASCL4 | ASH1L | ASMTL | ASPM | ASTN1 | ASXL1 | ASXL2 |
| ATM | ATP1A1 | ATP2B3 | ATP12A | ATR | ATRX | AURKA | AURKB | AXIN1 | AXIN2 |
| AXL | B2M | BACH1 | BAP1 | BARD1 | BAZ2B | BBC3 | BBS9 | BCAS1 | BCL2 |
| BCL2A1 | BCL2L1 | BCL2L2 | BCL2L11 | BCL3 | BCL6 | BCL9 | BCL10 | BCL11A | BCL11B |
| BCOR | BCORL1 | BCR | BIRC3 | BLM | BMPR1A | BMPR2 | BNC2 | BPTF | BRAF |
| BRCA1 | BRCA2 | BRD2 | BRD3 | BRD4 | BRIP1 | BRSK1 | BRWD1 | BTG1 | BTG2 |
| BTK | BUB1 | C1QA | C1S | C3orf70 | C7orf53 | C8orf34 | C11orf30 | C15orf23 | C15orf55 |
| CACNA1E | CADM2 | CALR | CAMTA1 | CARD11 | CASP1 | CASP8 | CASQ2 | CBFB | CBL |
| CBLB | CBR1 | CBR3 | CCDC168 | CCNA1 | CCNB3 | CCND1 | CCND2 | CCND3 | CCNE1 |
| CCT3 | CCT5 | CD5L | CD22 | CD33 | CD70 | CD74 | CD79A | CD79B | CD274 |
| CDA | CDC73 | CDH1 | CDH11 | CDH18 | CDH23 | CDK4 | CDK6 | CDK8 | CDK12 |
| CDK13 | CDKN1A | CDKN1B | CDKN2A | CDKN2B | CDKN2C | CEBPA | CFTR | CHD1 | CHD2 |
| CHD4 | CHD6 | CHD8 | CHD9 | CHEK1 | CHEK2 | CHFR | CHI3L1 | CHN1 | CIC |
| CIITA | CLDN18 | CLP1 | CNOT3 | CNOT4 | CNTN1 | CNTN5 | CNTNAP1 | CNTNAP5 | COL1A1 |
| COL2A1 | COL5A1 | COL5A2 | COL5A3 | COPS2 | CPS1 | CREBBP | CRIPAK | CRKL | CRLF2 |
| CRNKL1 | CRTC1 | CSF1 | CSF1R | CSF3R | CSMD1 | CSMD3 | CSNK1A1 | CSNK1G3 | CTCF |
| CTLA4 | CTNNA1 | CTNNA2 | CTNNB1 | CTNND1 | CUL3 | CUL4A | CUX1 | CXCR4 | CYBA |
| CYLD | CYP1A1 | CYP1B1 | CYP2A13 | CYP2C8 | CYP2D6 | CYP3A4 | CYP17A1 | CYP19A1 | DAXX |
| DCC | DDR1 | DDR2 | DDX3X | DDX5 | DEK | DHX9 | DHX35 | DICER1 | DIS3 |
| DIS3L2 | DLC1 | DMD | DNAH6 | DNAJB1 | DNM2 | DNMT3A | DNMT3B | DOCK2 | DOT1L |
| DPYD | DRGX | DTX1 | DUSP22 | DYSF | E2F3 | EBF1 | ECT2L | EED | EEF1A1 |
| EGFR | EGR3 | EIF1AX | EIF2AK3 | EIF2C3 | EIF3A | EIF4A2 | EIF4G3 | ELAC2 | ELF1 |
| ELF3 | ELMO1 | EME2 | EMID2 | EML4 | EP300 | EPAS1 | EPC1 | EPCAM | EPHA1 |
| EPHA2 | EPHA3 | EPHA4 | EPHA5 | EPHA6 | EPHA7 | EPHB1 | EPHB2 | EPHB4 | EPHB6 |
| EPOR | EPPK1 | EPS15 | ERBB2 | ERBB3 | ERBB4 | ERCC1 | ERCC2 | ERCC3 | ERCC4 |
| ERCC5 | ERG | ERRFI1 | ESR1 | ESR2 | ETS1 | ETV1 | ETV4 | ETV5 | ETV6 |
| EWSR1 | EXT1 | EXT2 | EZH2 | EZR | F8 | FAM5C | FAM46C | FAM123B | FAM131B |
| FAM135B | FAM157B | FAM175A | FANCA | FANCC | FANCD2 | FANCE | FANCF | FANCG | FANCI |
| FANCL | FANCM | FAP | FAS | FASLG | FAT1 | FAT2 | FAT3 | FAT4 | FBXW7 |
| FCGR1A | FCGR2A | FCGR2B | FCGR3A | FCRL4 | FGF3 | FGF4 | FGF6 | FGF7 | FGF10 |
| FGF12 | FGF14 | FGF19 | FGF23 | FGFR1 | FGFR2 | FGFR3 | FGFR4 | FH | FLCN |
| FLG | FLI1 | FLNC | FLT1 | FLT3 | FLT4 | FMN2 | FN1 | FNDC4 | FOXA1 |
| FOXA2 | FOXL2 | FOXO1 | FOXP1 | FOXQ1 | FRMPD4 | FUBP1 | FUS | FXR1 | FZD1 |
| G3BP2 | GAB1 | GAB2 | GABRA6 | GALNT12 | GATA1 | GATA2 | GATA3 | GATA4 | GATA6 |
| GEN1 | GFRAL | GID4 | GIGYF1 | GKN2 | GLB1L3 | GLI1 | GLI2 | GLI3 | GNA11 |
| GNA13 | GNAQ | GNAS | GNG2 | GPC3 | GPR124 | GPS2 | GPX1 | GRB7 | GRIN2A |
| GRM3 | GSK3B | GSTM5 | GSTP1 | GUSB | H3F3A | H3F3B | H3F3C | HCLS1 | HCN1 |
| HDAC1 | HDAC2 | HDAC4 | HDAC9 | HECW1 | HEY1 | HGF | HIST1H1C | HIST1H1D | HIST1H1E |
| HIST1H2AC | HIST1H2AG | HIST1H2AL | HIST1H2AM | HIST1H2BC | HIST1H2BD | HIST1H2BJ | HIST1H2BK | HIST1H2BO | HIST1H3B |
| HIST1H3C | HIST1H3D | HIST1H3F | HIST1H3G | HIST1H3H | HIST1H3I | HIST1H4I | HIST3H3 | HLA-A | HLA-B |
| HLA-C | HLF | HMCN1 | HNF1A | HNF1B | HNRPDL | HOXA3 | HOXA9 | HOXA11 | HOXA13 |
| HOXB13 | HOXC13 | HOXD11 | HOXD13 | HRAS | HSD3B1 | HSP90AA1 | HSP90AB1 | HSPA8 | HSPD1 |
| HSPH1 | ICK | ID3 | IDH1 | IDH2 | IFITM3 | IFNG | IFNGR1 | IGF1 | IGF1R |
| IGF2 | IGF2R | IGLL5 | IKBKE | IKZF1 | IKZF2 | IKZF3 | IL1RAPL1 | IL6 | IL6ST |
| IL7R | IL10 | IL21R | IMPG1 | ING1 | INHBA | INPP4A | INPP4B | INPPL1 | INSR |
| IRF2 | IRF4 | IRF6 | IRS1 | IRS2 | ITGB3 | ITK | ITSN1 | JAK1 | JAK2 |
| JAK3 | JUN | KALRN | KAT6A | KAT6B | KCNJ5 | KCNQ2 | KDM2B | KDM5A | KDM5C |
| KDM6A | KDR | KEAP1 | KEL | KIF5B | KIT | KLF4 | KLHL6 | KLK1 | KRAS |
| KRTAP5-5 | L3MBTL1 | LAMA2 | LATS1 | LATS2 | LCP1 | LEF1 | LIFR | LPHN2 | LPP |
| LRP1B | LRP2 | LRP4 | LRP5 | LRP6 | LRRC7 | LRRK2 | LTK | LYN | LZTS1 |
| MAD1L1 | MAF | MAGI2 | MAML2 | MAML3 | MAP2K1 | MAP2K2 | MAP2K4 | MAP3K1 | MAP3K13 |
| MAPK1 | MAPK3 | MAX | MC1R | MCC | MCL1 | MDC1 | MDM2 | MDM4 | MECOM |
| MED12 | MEF2B | MEF2C | MEN1 | MERTK | MET | MGA | MIB1 | MIOS | MITF |
| MKL1 | MKNK1 | MLH1 | MLH3 | MLL | MLL2 | MLL3 | MLL4 | MLLT3 | MMP2 |
| MMP11 | MN1 | MNDA | MNX1 | MPL | MRE11A | MS4A1 | MSH2 | MSH3 | MSH4 |
| MSH6 | MSN | MSR1 | MST1R | MTAP | MTHFR | MTOR | MTRR | MUTYH | MYB |
| MYC | MYCL1 | MYCN | MYD88 | MYH9 | MYH11 | MYH14 | MYO3A | MYOD1 | NAP1L1 |
| NAV3 | NBN | NCAM2 | NCOA3 | NCOA4 | NCOR1 | NCOR2 | NCSTN | NDUFA13 | NF1 |
| NF2 | NFATC4 | NFE2L2 | NFE2L3 | NFKBIA | NKX2-1 | NKX3-1 | NLRC3 | NOS3 | NOTCH1 |
| NOTCH2 | NOTCH3 | NOTCH4 | NPM1 | NQO1 | NR1I2 | NR2F2 | NR4A2 | NRAS | NRG1 |
| NRP2 | NRXN1 | NSD1 | NT5C2 | NTHL1 | NTM | NTRK1 | NTRK2 | NTRK3 | NUMA1 |
| NUP93 | NUP98 | NUP210 | OBSCN | OMD | OPCML | OR2T4 | OR4A15 | OR4C6 | OR5L2 |
| OR6F1 | OR11G2 | P2RY8 | P4HB | PABPC1 | PABPC3 | PAG1 | PAK1 | PAK3 | PAK7 |
| PALB2 | PARK2 | PARP1 | PARP2 | PARP3 | PASK | PAX3 | PAX5 | PAX7 | PBRM1 |
| PC | PCDH18 | PCK1 | PCSK6 | PCSK7 | PDCD1 | PDCD1LG2 | PDE4DIP | PDGFB | PDGFRA |
| PDGFRB | PDILT | PDK1 | PER1 | PGR | PHF6 | PIK3C2A | PIK3C2B | PIK3C2G | PIK3C3 |
| PIK3CA | PIK3CB | PIK3CD | PIK3CG | PIK3R1 | PIK3R2 | PIM1 | PKD1L2 | PKHD1 | PLAG1 |
| PLCB1  POLD1 | PLCG1  POLE | PLCG2  POLQ | PLEKHS1  POM121 | PLK1  POM121L12 | PLXNA1  POT1 | PLXNB2  PPARG | PMS1  PPM1D | PMS2  PPP1R17 | PNRC1  PPP2R1A |
| PPP2R2A | PPP6C | PRDM1 | PRDM16 | PREX2 | PRF1 | PRKAA1 | PRKAR1A | PRKCB | PRKCI |
| PRKDC | PRRX1 | PRSS1 | PRX | PSG2 | PSIP1 | PSMB1 | PSMB5 | PTCH1 | PTCH2 |
| PTEN | PTGS1 | PTGS2 | PTPN2 | PTPN11 | PTPRB | PTPRD | PTPRK | PTPRO | PTPRS |
| PTPRT | PTPRU | QKI | RAB35 | RAC1 | RAC2 | RAD21 | RAD50 | RAD51 | RAD51B |
| RAD51C | RAD51D | RAD52 | RAD54B | RAD54L | RAF1 | RANBP2 | RARA | RASGRP1 | RB1 |
| RBL1 | RBM10 | RECQL | RECQL4 | REL | RELA | RELN | RET | RFC1 | RGS3 |
| RHEB | RHOA | RHOH | RHOT1 | RICTOR | RINT1 | RIT1 | RNASEL | RNF43 | ROBO1 |
| ROBO2 | ROBO3 | ROCK1 | ROS1 | RPA1 | RPGR | RPL22 | RPS6KB1 | RPS6KB2 | RPS20 |
| RPTOR | RREB1 | RSPO2 | RSPO3 | RUNX1 | RUNX1T1 | RUNX2 | RXRA | RYR1 | RYR2 |
| SBDS | SCUBE2 | SDC4 | SDHA | SDHAF2 | SDHB | SDHC | SDHD | SEMA3A | SEMA3E |
| SEMA6A | SERPINA7 | SERPINB3 | SERPINB4 | SETBP1 | SETD2 | SETDB1 | SF1 | SF3B1 | SGCZ |
| SGK1 | SH2B3 | SH3PXD2A | SI | SIN3A | SLC1A2 | SLC16A1 | SLC22A2 | SLC22A3 | SLC22A16 |
| SLC34A2 | SLCO1B3 | SLIT1 | SLIT2 | SLX4 | SMAD2 | SMAD3 | SMAD4 | SMARCA4 | SMARCB1 |
| SMARCD1 | SMARCE1 | SMC1B | SMO | SNCAIP | SNTG1 | SNX29 | SOCS1 | SOD2 | SOS1 |
| SOX2 | SOX9 | SOX10 | SOX17 | SPEN | SPINK1 | SPOP | SPRR3 | SPSB4 | SPTA1 |
| SRC | SRD5A2 | SRGAP3 | SRSF2 | SRSF7 | STAG1 | STAG2 | STAT1 | STAT3 | STAT4 |
| STK11 | SUCLG1 | SUFU | SULT1A1 | SVEP1 | SYK | SYNCRIP | SYNE1 | TACSTD2 | TAF1 |
| TAF1L | TAF15 | TAL1 | TBL1XR1 | TBX3 | TBX15 | TBX22 | TCEB1 | TCF3 | TCF4 |
| TCF7L2 | TCF12 | TCL1A | TEC | TEK | TENM3 | TERC | TERT | TET1 | TET2 |
| TFDP1 | TFE3 | TGFBR1 | TGFBR2 | THBS2 | TIPARP | TJP1 | TLL2 | TLR4 | TLX3 |
| TMEM127 | TMEM132D | TMPRSS2 | TNFAIP3 | TNFRSF14 | TNFSF11 | TNN | TOP1 | TOP2A | TP53 |
| TP53BP1 | TP63 | TP73 | TPM3 | TPR | TRAF2 | TRAF7 | TRIM58 | TRIO | TRPC5 |
| TRRAP | TSC1 | TSC2 | TSHR | TSHZ2 | TSHZ3 | TTF1 | TUBA3C | TUBB3 | TUSC3 |
| TXNIP | TYMS | TYR | TYRO3 | U2AF1 | UBE2D2 | UBR5 | UGT1A1 | UMPS | UPF3B |
| USH2A | USP6 | USP8 | VEGFA | VEZF1 | VHL | VIM | VTCN1 | WASF3 | WDTC1 |
| WHSC1 | WHSC1L1 | WIPF1 | WNK1 | WNT5A | WRN | WSCD2 | WT1 | WWOX | WWP1 |
| WWP2 | XPC | XPO1 | XRCC1 | XRCC2 | XRCC3 | YAP1 | ZBTB16 | ZC3H11A | ZFHX3 |
| ZFP36L1 | ZFP36L2 | ZFPM2 | ZIC3 | ZMAT3 | ZNF217 | ZNF521 | ZNF638 | ZNF703 | ZNF750 |
| ZNF804B |  |  |  |  |  |  |  |  |  |

Supplementary Table 2. Sequence results of point mutation and insertion loss in tumor tissue

| Gene | Transcript | Base mutation | Amino acid mutation | Functional region | Mutation frequency |
| --- | --- | --- | --- | --- | --- |
| JUN | NM_002228.3 | c.466A>T | p.S156C | EX1 | 41.9% |
| EGFR | NM_005228.3 | c.3136A>T | p.T1046S | EX26 | 41.4% |
| ARID1A | NM_006015.4 | c.1741C>T | p.Q581* | EX3 | 38.2% |
| DLC1 | NM_182643.2 | c.568A>T | p.S190C | EX2 | 32.7% |
| CTNNB1 | NM_001904.3 | c.121A>G | p.T41A | EX3 | 28.6% |
| FANCC | NM_000136.2 | c.533A>T | p.E178V | EX7 | 27.3% |
| SOX17 | NM_022454.3 | c.392A>G | p.D131G | EX2 | 26.6% |
| SLX4 | NM_032444.2 | c.761-2A>T | . | IVS3 | 26.4% |
| BRD4 | NM_058243.2 | c.3674A>T | p.E1225V | EX18 | 25.4% |
| STAT3 | NM_139276.2 | c.1919A>T | p.Y640F | EX21 | 25.2% |
| MST1R | NM_002447.2 | c.550A>T | p.T184S | EX1 | 25.1% |
| KDR | NM_002253.2 | c.1817T>A | p.L606* | EX13 | 24.8% |
| PIK3C3 | NM_002647.2 | c.1021C>A | p.L341I | EX10 | 23.5% |
| TRIO | NM_007118.2 | c.2165A>T | p.Q722L | EX12 | 22.3% |
| KDR | NM_002253.2 | c.1966G>A | p.V656I | EX13 | 20.4% |
| DDR1 | NM_001954.4 | c.874A>T | p.T292S | EX10 | 20.2% |
| MAGI2 | NM_012301.3 | c.2653T>C | p.S885P | EX16 | 8.3% |
| XRCC2 | NM_005431.1 | c.49C>T | p.R17* | EX2 | 7.4% |

Supplementary Table 3. Sequence results of copy number variantion in tumor tissue

| Gene | Transcript | Type of variant | Functional region | Copy coefficient |
| --- | --- | --- | --- | --- |
| MYC | NM_002467.4 | amplification | All exon | 1.9 |
| CDKN2B | NM_004936.3 | loss | All exon | 0.5 |

Supplementary Table 4. Volumetry and growth kinetics after simultaneous TACE and PVE

|  | Initial rapid growth phase | Steady regrowth phase |
| --- | --- | --- |
| ESLV，cm^3^ | 1049.86 | 1049.86 |
| Start FLV, cm^3^ | 404 | 670.65 |
| End FLV, cm^3^ | 657.59 | 886.95 |
| Start FLV/ESLV | 38.4% | 63.8% |
| End FLV/ESLV | 62.6% | 84.4% |
| Increment in FLV/ESLV, % | 24.2% | 20.6% |
| Absolute increment in FLV, cm^3^ | 253.59 | 216.3 |
| Absolute increment in FLV , % | 62.7 | 32.2 |
| Increment in FLV per day, % | 2.23 | 1.15 |
| Rate of hypertrophy, cm^3^/d | 9.05 | 7.72 |
| Increment in FLV/ESLV per day, % | 0.86 | 0.74 |

ESLV, estimated standard liver volume; FLV, future liver remnant volume; TACE, transcatheter arterial chemoembolization; PVE, portal vein embolization.
