# Supplementary material for: Case Report: Successful conversion and salvage resection of huge hepatocellular carcinoma with portal vein tumor thrombosis and intrahepatic metastasis via sequential hepatic arterial infusion chemotherapy, lenvatinib plus PD-1 antibody followed by simultaneous transcatheter arterial chemoembolization, and portal vein embolization
Source: Front Immunol. 2023 Oct 18;14:1285296. doi: 10.3389/fimmu.2023.1285296 (PMC10622745; doi:10.3389/fimmu.2023.1285296)
Supplement: Supplementary file 3 [file Table_5.docx]

Supplementary Table 5. Members of Tongji multidisciplinary team for precision treatment of hepatobiliary cancer.

| Department | Name |
| --- | --- |
| Hepatic Surgery Center | Xiao-ping Chen, Bi-xiang Zhang, Wan-guang Zhang, Peng Zhu, Bin-hao Zhang, Ze-yang Ding, Zhan-guo Zhang, Xin Luo, Jian-ping Zhao |
| Oncology | Hong Qiu, Yan-mei Zou, Qiang Fu, Liang Zhuang, Hua Xiong, Li-hong Zhang, Shun-fang Liu, Li Sun |
| Pathology | Dong Kuang, Xu-guang Liu |
| Radiology | Zhen Li, Ya-qi Shen, Xue-mei Hu, Yan Luo |
| Interventional Radiology | An-hui Xu, Nan Wang, Ke-tao Mu |
| Medical Ultrasound | Kai-yan Li, Hong-chang Luo, Shu Zhu, Wei Zhang, Jing-yuan Chen, Shu-jun Yang |
| Nuclear Medicine | Dong-yi Wan |
| Gastrointestinal Surgery & GI Cancer Research Institute | Gui-hua Wang |
| Gastroenterology | Mei Liu, Qian Chen, Hua Qin, Bo Wang, Li He |
| Institute of Infectious Disease | Wei Guo, Ming Ni |
